# The Effectiveness of Time-Restricted Eating as an Intermittent Fasting Approach on Shift Workers’ Glucose Metabolism: A Systematic Review and Meta-Analysis

**DOI:** 10.3390/nu17101689

**Published:** 2025-05-15

**Authors:** Jia Ying Jennell Koh, Celine Yu Han Tan, Meng Li, Mei Hui Liu, Han Shi Jocelyn Chew

**Affiliations:** 1 Alice Lee Centre for Nursing Studies, Yong Loo Lin School of Medicine, National University of Singapore, Singapore 117543, Singapore; jennell.koh.jy@u.nus.edu (J.Y.J.K.); celine.tyh@u.nus.edu (C.Y.H.T.); 2 Department of Endocrinology and Metabolism, First Affiliated Hospital of Xi’an Jiaotong University, Xi’an 710061, China; limeng2000@126.com; 3 Department of Food Science and Technology, Faculty of Science, National University of Singapore, Singapore 117543, Singapore; fstlmh@nus.edu.sg; 4 Cardiovascular Metabolic Disease Translational Research Programme, Yong Loo Lin School of Medicine, National University of Singapore, Singapore 117543, Singapore

**Keywords:** time-restricted eating, intermittent fasting, circadian rhythms, shift work, glucose metabolism, cardiometabolic health, chrononutrition

## Abstract

**Background/Objectives**: Shift workers face higher risks of impaired glucose metabolism due to irregular eating habits and circadian misalignment. Time-restricted eating (TRE) could improve glucose metabolism by aligning food intake with the circadian clock, but its effectiveness remains unclear. **Methods**: Ten electronic databases (PubMed, EMBASE, Cochrane Library, CINAHL, PsycINFO, Scopus, Web of Science, ProQuest Dissertations and Theses, Science.gov, and ClinicalTrials.gov) were searched from journal inception to September 2024. Only randomized controlled trials (RCTs) involving shift workers were included. Meta-analyses with sensitivity analyses were conducted using a random-effects model to pool glucose metabolism and sleep outcomes, with heterogeneity and quality assessments performed. **Results**: Six RCTs were included. TRE demonstrated positive but non-significant effects on glucose metabolism outcomes: fasting blood glucose (weighted mean difference [WMD]: −0.02 mmol/L, 95% confidence interval [CI]: −0.13 to 0.10, *I*^2^ = 0%), fasting blood insulin (WMD: −5.77 pmol/L, 95% CI: −85.62 to 74.08, *I*^2^ = 92%), HOMA-IR (WMD: −0.50, 95% CI: −2.76 to 1.76, *I*^2^ = 82%), 2 h postprandial glucose (WMD: −0.65 mmol/L, 95% CI: −3.18 to 1.89, *I*^2^ = 86%), total sleep time (*g* = 0.07, 95% CI: −0.23 to 0.37, *I*^2^ = 0%), and sleep efficiency (*g* = −0.05, 95% CI: −0.63 to 0.53, *I*^2^ = 62%). Sensitivity analyses yielded similar findings, and overall certainty of evidence was rated ‘very low’. **Conclusions**: While TRE shows potential for improving the glucose metabolism in shift workers, current evidence remains inconclusive due to small sample sizes and study limitations. Future research should prioritize well-powered TRE RCTs in shift workers that adhere to a 6–10 h eating window. Incorporating early-TRE schedules with sleep hygiene may optimize metabolic outcomes, with circadian biomarkers analyzed to better elucidate the mechanistic pathway implicated.

## 1. Introduction

Almost a quarter of the global workforce engages in shift work [1], a work schedule that has been associated with detrimental cardiometabolic health outcomes such as obesity, impaired glucose control and dyslipidemia [2,3,4,5]. Among these outcomes, impaired glucose metabolism is of primary concern, given its central role in the development of type 2 diabetes and its interconnectedness with multiple physiological systems [6]. If left unregulated, impaired glucose metabolism may not only exacerbate cardiometabolic disease (CMD) risk but could contribute to broader systemic dysfunction [7]. While the underlying mechanisms are not fully understood, some researchers posit that circadian misalignment may be a key contributing factor [5,8].

Circadian rhythm refers to the body’s natural, internal process that regulates the sleep–wake cycle and other physiological processes in a roughly 24 h cycle [9]. Circadian rhythms affect sleep patterns, hormone release (e.g., melatonin and cortisol), body temperature, and metabolism, playing a crucial role in overall health and well-being [10]. The circadian clock is made up of two parts: the (1) central clock that is based in the brain’s hypothalamic suprachiasmatic nucleus (SCN) and regulates the timing of various physiological functions based on light as a cue; and the (2) peripheral clock that is present in various bodily tissues and regulates the timing of various physiological functions based on food intake, temperature and exercise as cues [9,10,11].

When shift workers are forced to adapt their bodies to light at night, they experience circadian misalignment where exposure to light conflicts with the body’s natural metabolic adaptations to darkness [8]. This results in a disruption of the clock gene function, leading to impaired beta cell function, irregular insulin production, insulin resistance and impaired glucose metabolism [12,13]. Shift workers have also been shown to exhibit higher 24 h insulin and leptin levels [14], postprandial glucose and lipid measurements [15,16]. These outcomes may be compounded by late meal timings, which can desynchronize peripheral clocks from the circadian rhythm, further exacerbating circadian misalignment and impairing metabolic regulation in shift workers [10,17].

While it may be difficult to counteract or remove light to prevent circadian misalignment, one could reduce the effects of such by aligning eating patterns with the circadian rhythm. Nighttime fasting, a form of intermittent fasting that restricts food consumption to the daytime while fasting at night, has recently been proposed to reduce circadian misalignment in shift workers [18]. More specifically, time-restricted eating (TRE), a more regulated and structured form of nighttime fasting, has become an area of growing interest due to its potential metabolic benefits and its focus on the circadian alignment of food intake. TRE involves restricting all food consumption to the daytime (active phase), typically within a 6–10 h window, while fasting takes place at night (inactive phase), thereby aligning eating patterns with the circadian rhythm [19]. TRE stands out from other dietary interventions, as it focuses on when food is consumed, rather than the amount, type, or composition of food eaten [11]. While TRE is still a relatively novel dietary approach, emerging research has shown that it improves the glucose metabolism by enhancing insulin sensitivity and lowering glycated hemoglobin (HbA1c) and FBG levels across various metabolic profiles [20,21,22]. However, most evidence was derived from animal studies [23,24] and existing review articles were primarily narrative [18,25,26,27,28] or mechanistic [15,29,30] in nature. Few systematic reviews and meta-analyses have explored these associations [31,32,33], with three meta-analyses suggesting that TRE may benefit cardiometabolic health among the general population, although findings remain inconsistent [34,35,36]. To the best of the authors’ knowledge, no systematic reviews or meta-analyses have specifically focused on the impact of TRE on the glucose metabolism of shift workers, a group at higher risk of circadian misalignment and impaired glucose metabolism. Only one narrative review has examined this association, focusing, however, on establishing nutritional guidelines [18].

Hence, this systematic review and meta-analysis was undertaken to evaluate the effectiveness of TRE on shift workers’ glucose metabolism. Given the pivotal role of glucose metabolism in CMD progression [37], its sensitivity to circadian rhythms [38], and its interconnectedness with multiple physiological systems [6], focusing on this outcome provides a targeted insight into TRE’s potential benefits, while informing future research on its relationship with CMDs.

## 2. Materials and Methods

This systematic review with meta-analysis was conducted according to the Preferred Reporting Items for Systematic Reviews and Meta-Analyses (PRISMA) guidelines [39] (Supplementary Table S1) and registered with the International Prospective Register of Systematic Reviews (PROSPERO) (registration number: CRD42024616868). Inter-rater reliability was calculated for study selection, methodological quality, and overall certainty of evidence between 2 independent reviewers (J.Y.J.K. and C.Y.H.T.) using Cohen’s kappa (κ) statistics: no agreement (κ = 0), slight agreement (κ = 0.01–0.20), fair agreement (κ = 0.21–0.40), moderate agreement (κ = 0.41–0.60), substantial agreement (κ = 0.61–0.80), and perfect agreement (κ = 0.81–1.00) [40].

### 2.1. Eligibility Criteria

Studies were included if they (1) involved individuals in a simulated or free-living shift work environment; (2) included an intervention group that followed a TRE regimen with overnight fasting, but not necessarily limited to a 6–10 h eating window; (3) had a passive control group allowing unrestricted overnight eating; (4) assessed at least one of the following glucose metabolism indicators: FBG, fasting blood insulin, 2 h postprandial glucose following breakfast, or the Homeostatic Model Assessment of Insulin Resistance (HOMA-IR). Secondary outcomes included total sleep time and sleep efficiency following a night shift; (5) were randomized controlled trials (RCTs); and (6) were published in English in internationally refereed journals, theses, or full-text conference papers.

Studies were excluded if they (1) involved non-night-shift workers, individuals with acute or chronic diseases, or animal models; (2) included other fasting protocols that do not align with the body’s circadian rhythm (e.g., alternate day fasting, Ramadan fasting); (3) combined TRE with other interventions; (4) were non-RCTs or secondary research; and (5) were protocols, abstract-only papers, summaries, expert commentaries, or books (Supplementary Table S2).

### 2.2. Search Strategy

A three-step search strategy was conducted by J.Y.J.K. in September 2024 [41]. The first step included an initial search carried out on PubMed and Google Scholar to compile a list of keywords and controlled vocabulary, to ensure all relevant studies were included. Furthermore, a preliminary search on PROSPERO was conducted to prevent the duplication of this review.

Secondly, the list of keywords and controlled vocabulary was used to perform a comprehensive search using seven electronic databases (PubMed, EMBASE, The Cochrane Library, CINAHL, PsycINFO, Scopus, Web of Science). The search strategy combined controlled vocabulary and free-text keywords, using Boolean operators AND/OR, following the population, intervention, and outcome framework. Search terms included population (‘shift work’, ‘night shift’), intervention (‘time-restricted eating’, ‘intermittent fasting’), and outcome (‘insulin’, ‘glucose’) (Supplementary Table S3).

The last step involved searching for gray literature on ProQuest Dissertations and Theses, Science.gov, and ClinicalTrials.gov. Reference lists of all included studies and existing systematic reviews were also hand-searched for additional studies. No limit was set on the date of publication to include all relevant studies. A specialist medical librarian was consulted to validate the search strategy. During the process of writing this manuscript, additional relevant studies published after September 2024 were also included.

### 2.3. Screening and Eligibility

All search results were imported into EndNote 21, where duplicates were removed using the software and manually checked. The remaining citations were transferred into Rayyan for title, abstract and full-text screening using the eligibility criteria. J.Y.J.K. screened the title and abstract, and both J.Y.J.K. and C.Y.H.T. independently reviewed the full-text articles and reference lists to ensure all relevant studies were included. Discrepancies were resolved by a third reviewer (H.S.J.C.).

### 2.4. Data Extraction

Data extraction was performed by J.Y.J.K. using a modified version of the Cochrane Handbook data extraction form [42]. This included information such as study details, methods used, participant characteristics, intervention and control group characteristics, and immediate post-intervention outcome measurements. Fasting and feeding windows were not always explicitly defined in some studies; however, they could be inferred based on the described protocols. WebPlotDigitizer version 5.2 was used to extract the mean and standard deviation (SD) of outcomes represented in graphical form when tabular data were unavailable, with a margin of error of ±0.1 [43] (Supplementary Table S4). In cases where studies assessed postprandial glucose at longer time points, data was extracted at the 2 h mark to maintain consistency across studies. For studies reporting age and BMI separately for each study group, weighted mean and pooled SD were calculated to summarize participant characteristics using the following formulas:

$$\mathrm{Weighted}\mathrm{mean}=\frac{\sum \left(w_{i}\cdot x_{i}\right)}{\sum w_{i}}$$

$${SD}_{pooled}=\sqrt{\frac{\sum \left(n_{i}-1\right)\cdot {SD}_{i}^{2}}{\sum \left(n_{i}-1\right)}}$$

where w_i_ is the weight, x_i_ is the mean, n_i_ is the sample size, and SD_i_ is the standard deviation of each group [42].

If missing or questionable data were identified, researchers were contacted via email (Supplementary Table S8, Supplementary Figure S1). This data extraction form has been piloted by J.Y.J.K. and C.Y.H.T. in 5 articles and deemed to be adequate.

### 2.5. Methodological Quality Appraisal

J.Y.J.K. and C.Y.H.T. independently assessed the risk of bias for each study using Cochrane’s Risk of Bias-1 (RoB-1) [44]. This tool assessed domains including random sequence generation, allocation concealment, blinding of participants and personnel, blinding of outcome assessment, incomplete outcome data, and selective outcome reporting, assessed on a rating of low, unclear, or high risk of bias. Discrepancies were resolved by a third reviewer (H.S.J.C.).

### 2.6. Overall Evidence Grading

The Grading of Recommendations, Assessment, Development, and Evaluation (GRADE) was used to determine the overall confidence in findings. Using GRADEpro GDT [45], J.Y.J.K. and C.Y.H.T. independently assessed articles on their study design, risk of bias, inconsistency, indirectness, imprecision, publication bias, and the importance of the outcome, rating evidence certainty as ‘very low’, ‘low’, ‘moderate’, or ‘high’ [46]. Disagreements were resolved by a third reviewer (H.S.J.C.).

### 2.7. Data Analysis

Meta-analyses were conducted using RevMan version 8.14.0. Units were first standardized across effect sizes to be pooled (Supplementary Table S5). A confidence interval (CI) of 95% or standard error (SE) were converted to SD using RevMan’s built-in calculator. For change-from-baseline data, post-intervention data were calculated by adding the change value to baseline data. Four outcomes were estimated using weighted mean difference (WMD) and two using standardized mean difference (SMD). SMD was used due to differences in measurement methods and expressed as Hedges’ *g* to account for the small number of studies in the meta-analyses [47]. Effect sizes were rated as small (0.2), medium (0.5), and large (0.8) [47].

Effect sizes were pooled using a random-effects model with generic inverse variance to address heterogeneity between studies and minimize imprecision of pooled effect estimates [48]. CIs of 95% were calculated using the Hartung–Knapp–Sidik–Jonkman or Wald-type methods, depending on the number of included studies in the meta-analysis and the presence of heterogeneity. The Hartung–Knapp–Sidik–Jonkman method, which uses t-distribution, was applied when there were at least 3 studies, and the heterogeneity estimate > 0. In other scenarios (i.e., 2 studies or heterogeneity estimate = 0), the Wald-type method with z-distribution was used [49]. Overall estimates were considered statistically significant at *p* < 0.05. Sensitivity analyses were conducted on FBG, fasting blood insulin, HOMA-IR, and 2 h postprandial glucose to assess result robustness, especially in the presence of considerable heterogeneity [48].

Heterogeneity was assessed and quantified using Cochran’s Q test and *I*^2^ statistic, where *p* < 0.1 indicated significance [48]. *I*^2^ values of 0–40% indicated low heterogeneity, 30–60% moderate, 50–90% substantial, and 75–100% represented considerable heterogeneity [48]. Restricted maximum likelihood was used to estimate heterogeneity variances due to its ability to handle few studies [49].

Results were illustrated using a forest plot.

Given the small number of studies included (*n* = 6), funnel plot and subgroup analyses could not be reliably conducted [48,50]. A narrative synthesis of study findings was conducted when quantitative synthesis was not feasible.

## 3. Results

### 3.1. Study Selection

**Figure 1:**
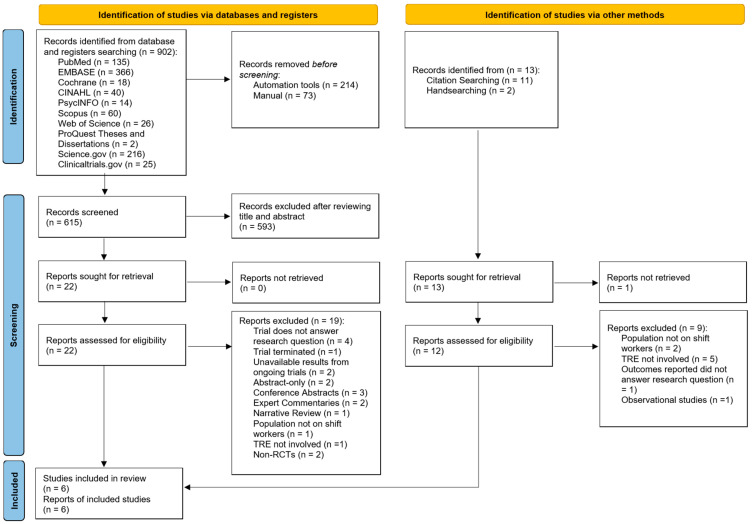
Preferred Reporting Items for Systematic Reviews and Meta-Analyses, 2020 (PRISMA 2020) flowchart for study selection [39].

A total of 902 articles were identified from electronic database and register searches (Figure 1). After removing duplicates, 615 articles were screened by title and abstract. Of these, 593 articles were excluded, leaving 22 for full-text screening. After further exclusions based on the eligibility criteria, three studies were included. An additional 13 articles were identified through citation searching and handsearching, with three meeting the inclusion criteria after full-text screening. Hence, a total of six articles were included in this review [51,52,53,54,55,56]. Inter-rater reliability for full-text selection between two independent reviewers was in perfect agreement (κ = 0.93).

### 3.2. Characteristics of Included Studies

Table 1 presents the characteristics of the six included studies (detailed table available in Supplementary Table S6), which involved 316 participants, conducted between 2021 and 2024 across the USA [52,54], Australia [53], New Zealand [51], The Netherlands [55] and Brazil [56]. Three studies employed parallel-group designs [51,52,54] and three used crossover designs [53,55,56]. Additionally, two studies utilized three-arm designs [51,56] and one was conducted as a pilot study [53].

Four studies examined healthy participants [51,52,55,56], one focused solely on individuals with abdominal obesity [53], and another included firefighters, both healthy and with potential cardiovascular disease (CVD) risk factors [54]. Free-living shift work studies included night shift workers, firefighters, nurses, and police officers [53,54,55,56], while simulated shift work studies investigated non-shift working adults [51,52]. The mean participant age ranged between 24.5 and 41 years old, and mean BMI ranged between 22.7 and 30.7, excluding one study where mean BMI was not reported [54]. A total of 186 males and 121 females were included in this review.

Study duration ranged from six days [51] to 14 weeks [54]. Different protocols were used across studies, with five studies following nighttime fasting protocols, with fasting: feeding ratios of 11.5:12.5 [51], 9.4:14.6 (converted from 28 h cycle) [52], 5:19 [53], 10:14 [55], and 7.5:16.5 [56], and one study following a strict TRE protocol with a fasting: feeding ratio of 14:10 [54].

### 3.3. Methodological Quality

**Figure 2:**
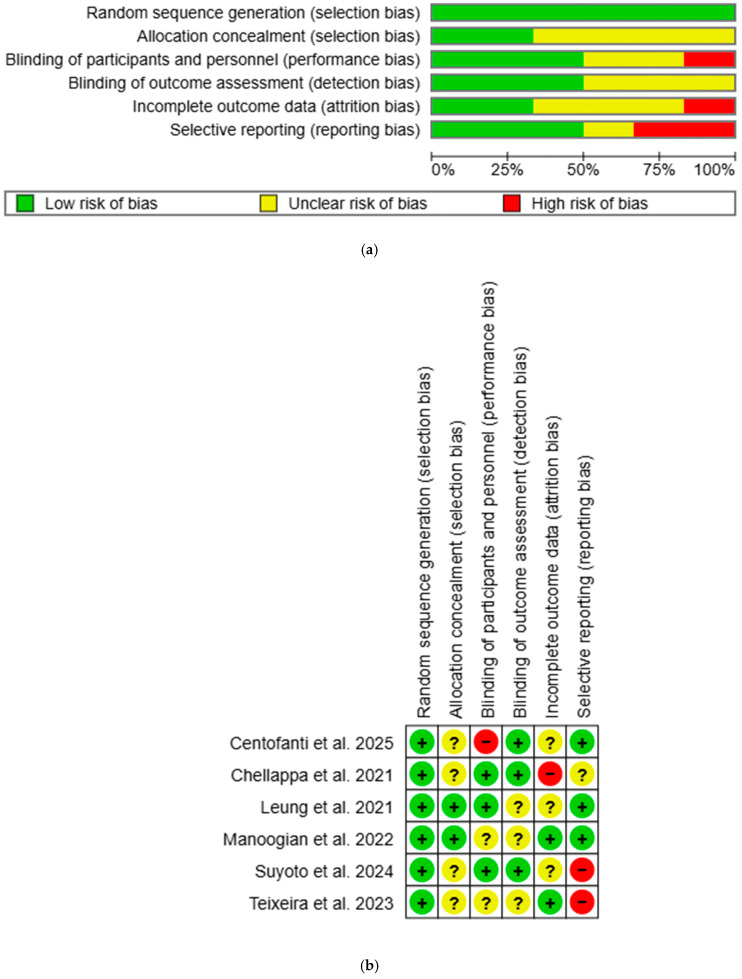
(**a**) Risk-of-bias graph using Cochrane RoB-1. “+” = low risk of bias, “?” = unclear risk of bias, and “−“ = high risk of bias. (**b**) Risk-of-bias summary using Cochrane RoB-1. “+” = low risk of bias, “?” = unclear risk of bias, and “−“ = high risk of bias [51,52,53,54,55,56].

Overall, most studies had low or unclear risk of bias, with a few studies showing high risk in certain domains (Figure 2). All studies were rated low risk for selection bias in random sequence generation. Two studies were rated low risk for selection bias in allocation concealment: one used opaque sealed envelope [53], while the other used third-party allocation concealment [54]. One study was rated high risk for performance bias as participants were aware of the group allocation [51], while three had low risk as single blinding was used [52,53,55]. Detection bias was rated low risk in three studies due to blinded outcome assessors [51] or objective outcome measurements [52,55]. Two studies had low risk for attrition bias: one used intention-to-treat (ITT) analysis [54], while the other had no attrition [56]. One study was rated high risk due to unaddressed missing data [52]. Reporting bias was high in two studies as published study protocols failed to report all outcomes [55,56]. All unclear risk-of-bias ratings were due to insufficient information to permit judgement. Inter-rater reliability for methodological quality between two independent reviewers was in perfect agreement (κ = 0.84).

### 3.4. Overall Certainty of Evidence

The overall certainty of evidence for all outcomes was rated ‘very low’ (Supplementary Table S7). All included studies were RCTs, with serious risk of bias due to unclear or high risk of bias in multiple domains. Inconsistency was rated ‘very serious’ for fasting blood insulin, HOMA-IR, and 2 h postprandial glucose due to high heterogeneity and non-overlapping confidence intervals. Indirectness was rated ‘not serious’ for all outcomes, as the population, intervention, control, and outcome of each study aligned with the research question. Imprecision was rated ‘very serious’ for FBG, fasting blood insulin, and HOMA-IR due to small sample sizes and wide confidence intervals, and ‘serious’ for 2 h postprandial glucose, total sleep time, and sleep efficiency due to small sample sizes. Publication bias was strongly suspected in all outcomes as funnel plot analysis could not be reliably conducted [50]. Inter-rater reliability for overall evidence grading between two independent reviewers was in perfect agreement (κ = 0.87).

### 3.5. Outcomes

#### 3.5.1. Fasting Blood Glucose

**Figure 3:**
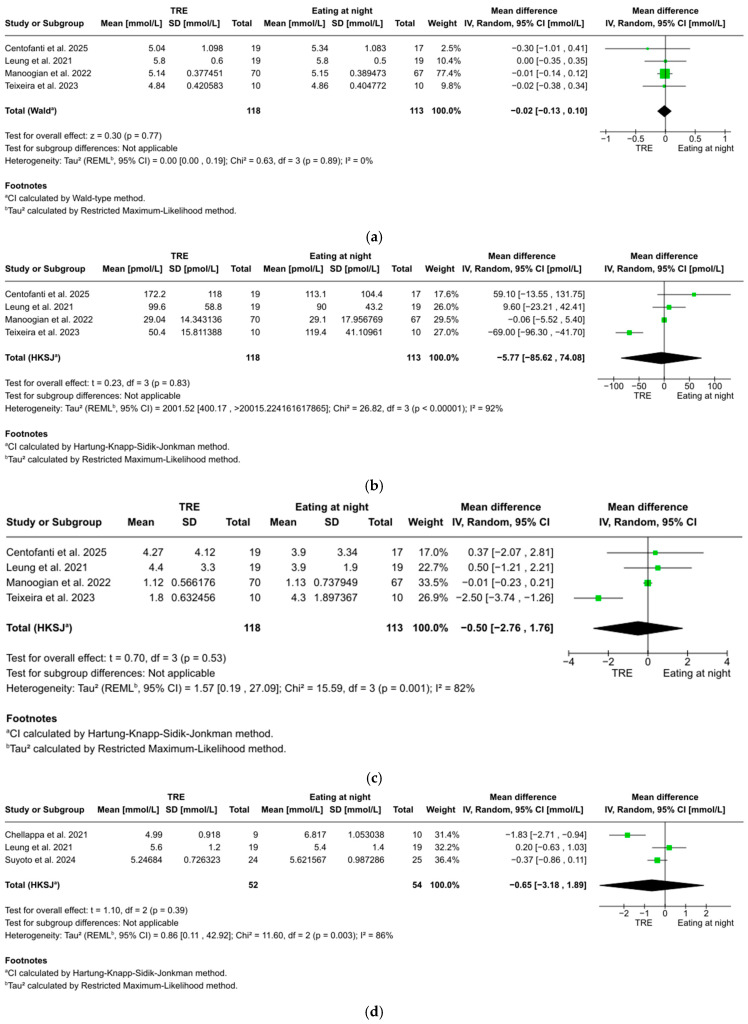

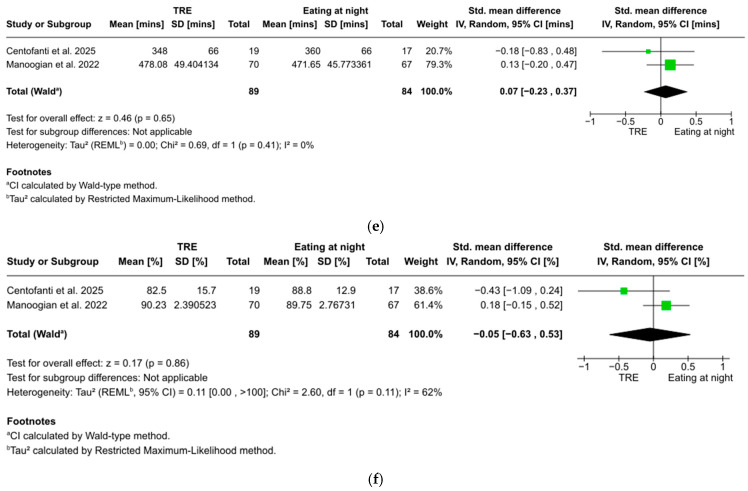
(**a**). Forest plot of the effect of TRE on fasting blood glucose. (**b**) Forest plot of the effect of TRE on fasting blood insulin. (**c**) Forest plot of the effect of TRE on HOMA-IR. (**d**) Forest plot of the effect of TRE on 2 h postprandial glucose. (**e**) Forest plot of the effect of TRE on total sleep time. (**f**) Forest plot of the effect of TRE on sleep efficiency [51,52,53,54,55,56].

FBG was reported in four studies with 202 participants, showing a non-significant trend favoring TRE over eating at night (WMD: −0.02 mmol/L, 95% CI: −0.13 to 0.10, *z* = 0.30, *p* = 0.77). No significant heterogeneity was found between studies (*I*^2^ = 0%, *p* = 0.89) (Figure 3a).

#### 3.5.2. Fasting Blood Insulin

Fasting blood insulin was reported in four studies with 202 participants, showing no statistically significant difference between TRE and eating at night (WMD: −5.77 pmol/L, 95% CI: −85.62 to 74.08, *t* = 0.23, *p* = 0.83). Considerable heterogeneity was found between studies (*I*^2^ = 92%, *p* < 0.001) (Figure 3b).

#### 3.5.3. HOMA-IR

HOMA-IR was reported in four studies with 202 participants, showing no statistically significant difference between TRE and eating at night (WMD: −0.50, 95% CI: −2.76 to 1.76, *t* = 0.70, *p* = 0.53). Considerable heterogeneity was found between studies (*I*^2^ = 82%, *p* = 0.001) (Figure 3c).

#### 3.5.4. Two-Hour Postprandial Glucose

Two-hour postprandial glucose was reported in three studies with 63 participants, showing no statistically significant difference between TRE and eating at night (WMD: −0.65 mmol/L, 95% CI: −3.18 to 1.89, *t* = 1.10, *p* = 0.39). Considerable heterogeneity was found between studies (*I*^2^ = 86%, *p* = 0.003) (Figure 3d).

#### 3.5.5. Total Sleep Time

Total sleep time was reported in two studies with 173 participants, showing no statistically significant effect and negligible effect size of TRE on total sleep time (*g* = 0.07, 95% CI: −0.23 to 0.37, *z* = 0.46, *p* = 0.65). No significant heterogeneity was found between studies (*I*^2^ = 0%, *p* = 0.41) (Figure 3e).

#### 3.5.6. Sleep Efficiency

Sleep efficiency was reported in two studies with 173 participants, showing no statistically significant effect and negligible effect size of TRE on sleep efficiency (*g* = −0.05, 95% CI: −0.63 to 0.53, *z* = 0.17, *p* = 0.86). Substantial heterogeneity was found between studies (*I*^2^ = 62%, *p* = 0.11) (Figure 3f).

#### 3.5.7. Sensitivity Analyses

**Figure A1:**
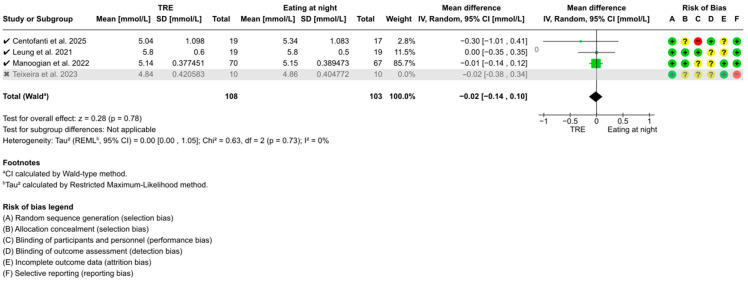
Sensitivity analysis of TRE on fasting blood glucose. “✓” = included in sensitivity analysis, “✗” = excluded in sensitivity analysis, “+” = low risk of bias, “?” = unclear risk of bias, and “−“ = high risk of bias [51,53,54,56].

**Figure A2:**
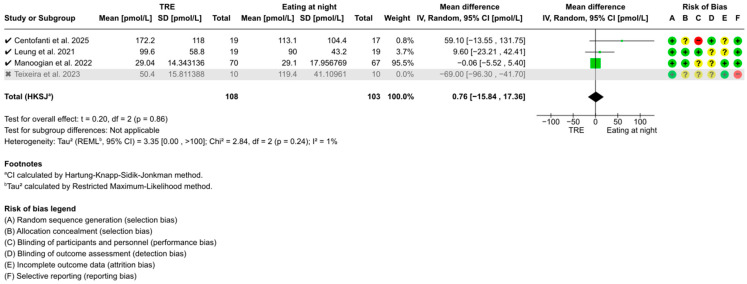
Sensitivity analysis of TRE on fasting blood insulin. “✓” = included in sensitivity analysis, “✗” = excluded in sensitivity analysis, “+” = low risk of bias, “?” = unclear risk of bias, and “−“ = high risk of bias [51,53,54,56].

**Figure A3:**
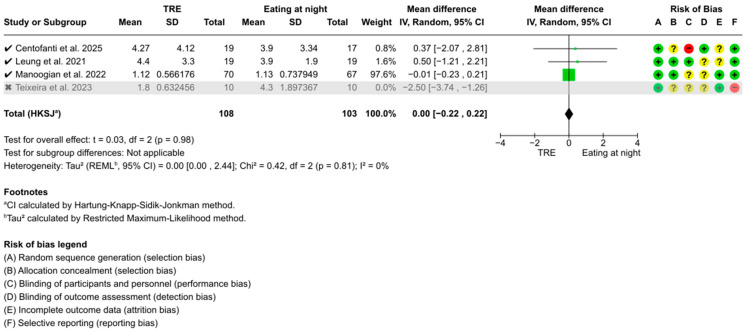
Sensitivity analysis of TRE on HOMA-IR. “✓” = included in sensitivity analysis, “✗” = excluded in sensitivity analysis, “+” = low risk of bias, “?” = unclear risk of bias, and “−“ = high risk of bias [51,53,54,56].

Sensitivity analyses identified Teixeira et al. [56] as a potential outlier due to its 4–7 h fasting duration before fasting level assessments, below the recommended ≥8 h [57]. Excluding this study revealed no significant changes in the overall conclusions of FBG (WMD: −0.02 mmol/L, 95% CI: −0.14 to 0.10, *z* = 0.28, *p* = 0.78) (Figure A1), fasting blood insulin (WMD: 0.76 pmol/L, 95% CI: −15.84 to 17.36, *t* = 0.20, *p* = 0.86) (Figure A2), or HOMA-IR (WMD: 0.00, 95% CI: −0.22 to 0.22, *t* = 0.03, *p* = 0.98) (Figure A3). However, heterogeneity for fasting blood insulin decreased from 92% (*p* < 0.001) to 1% (*p* = 0.24), and from 82% (*p* = 0.001) to 0% (*p* = 0.81) for HOMA-IR.

**Figure A4:**
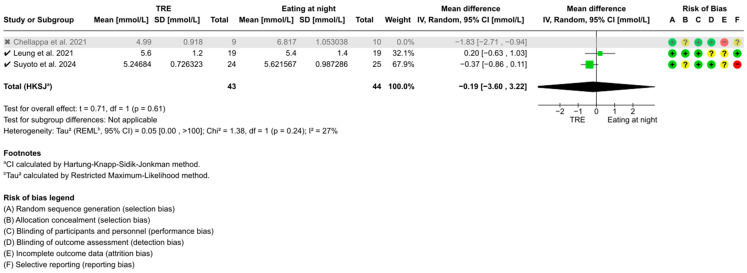
Sensitivity analysis of TRE on 2 h postprandial glucose. “✓” = included in sensitivity analysis, “✗” = excluded in sensitivity analysis, “+” = low risk of bias, “?” = unclear risk of bias, and “−“ = high risk of bias [52,53,55].

Additionally, Chellappa et al. [52] was identified as a potential outlier due to its 28 h circadian misalignment protocol, compared to the standard 24 h cycle. Excluding this study revealed no significant changes to the overall conclusion of 2 h postprandial glucose (WMD: −0.19 mmol/L, 95% CI: −3.60 to 3.22, *t* = 0.71, *p* = 0.61) (Figure A4). However, heterogeneity decreased from 86% (*p* = 0.003) to 27% (*p* = 0.24).

A sensitivity analysis for sleep efficiency was not feasible, as only two studies were included. The substantial heterogeneity observed could be attributed to variations in shift work settings, TRE schedules, and participant demographics. Further research is needed to clarify TRE’s effect on sleep efficiency and confirm these findings.

#### 3.5.8. Narrative Summary

Teixeria et al. [56] found no significant differences in FBG between TRE and eating-at-night groups, though fasting blood insulin and HOMA-IR were significantly lower in the TRE group. Chellappa et al. [52] found 3 h postprandial glucose (as reported) to be significantly increased in the eating-at-night group compared to baseline.

## 4. Discussion

### 4.1. Summary of Findings

To the best of the authors’ knowledge, this is the first systematic review that provided a comprehensive synthesis and appraisal of evidence on the effectiveness of TRE as an intermittent fasting approach on shift workers’ glucose metabolism. Six RCTs were selected, and meta-analyses were performed for primary (FBG, fasting blood insulin, HOMA-IR, 2 h postprandial glucose) and secondary outcomes (total sleep time, sleep efficiency). All meta-analyses yielded statistically non-significant results, with FBG demonstrating a trend favoring TRE, while other outcomes remained inconclusive due to the inability to pool data. Sensitivity analyses revealed no significant changes to the overall outcomes. These findings should be interpreted with caution, as further research is needed for definitive conclusions.

Several factors may explain the lack of statistical significance and inconclusive findings. First, small sample sizes in the included studies likely increased the risk of type II errors [58]—a common limitation in TRE studies due to its resource-intensive nature [34,36]. Additionally, the small number of studies included reduced the statistical power of pooled results, making it harder to detect a true effect [59]. Second, fasting durations may have been too short to trigger the metabolic switch from glucose to fat utilization through ketosis [60], which typically occurs after 12 h [61]. Only one study had a 14 h fasting period [54], suggesting that insufficient fasting durations in the other studies may have diluted TRE’s impact on glucose metabolism, introducing inconsistencies and limiting data pooling. Third, variability in how participant adherence to meal protocols was assessed may have introduced heterogeneity. While some studies employed digital tools such as mobile applications to track adherence [54,55], one relied on more subjective methods, such as self-reported dietary recalls [53], and one did not report adherence at all [56] (Supplementary Table S6). These differences in tracking methods, especially the use of subjective methods, may have introduced human error and reporting bias, contributing to heterogeneity and complicated data pooling, which could have led to inconclusive findings. Lastly, variability in shift schedules and participant demographics may have contributed to significant heterogeneity, reducing the statistical power of the analysis, limiting data pooling [62].

It should also be noted that despite our search terms being based on ‘time-restricted eating’ and ‘intermittent fasting’, the studies included in this review are predominantly focused on nighttime fasting protocols rather than strict TRE regimens. Throughout this review, only one study implemented a clearly defined TRE protocol [54], while the rest followed nighttime fasting protocols [51,52,53,55,56]. Intermittent fasting is an overarching term encompassing various voluntary dietary regimens, such as nighttime fasting, that cycle between periods of eating and fasting [60,63]. Nighttime fasting, which involves fasting during the night, might not always follow a clearly defined eating window, whereas TRE is a more specific form of nighttime fasting which emphasizes the circadian alignment of food intake and typically involves a regulated and consistent eating window, often between 6 and 10 h, and a fasting window of more than 12 h [19]. This distinction is important to make, as the less structured nature of nighttime fasting protocols in this study may have contributed to shorter fasting durations, significant heterogeneity, and inconclusive results. As such, the term TRE should be interpreted with discretion in the context of this review, as it encompasses both TRE and nighttime fasting protocols in the analyzed studies. Further studies focusing specifically on TRE protocols, with clearly defined and consistent eating and fasting windows, would help clarify its effects on shift workers.

### 4.2. Glucose Regulation (FBG, 2 Hour Postprandial Glucose, Fasting Blood Insulin, HOMA-IR)

Consistent with previous studies, TRE demonstrated a trend towards lower FBG, although the results differed in statistical significance [32,34,36]. Findings for 2 h postprandial glucose were inconclusive; however, previous studies have demonstrated that TRE significantly reduced postprandial glucose in overweight/obese men [64] and healthy older adults [65], and led to a 5% reduction in healthy adults [66]. Similarly, findings for fasting blood insulin and HOMA-IR were inconclusive, in line with two studies [34,67]. Nonetheless, three studies reported significant reductions in fasting blood insulin and HOMA-IR among individuals with varying metabolic profiles [35,68,69]. While these findings highlight TRE’s potential to improve glucose metabolism, inconsistencies across studies and participant heterogeneity underscores a need for further research, especially with larger sample sizes and among shift workers.

Despite these limitations, growing interest in TRE’s potential benefits are thought to be closely linked to the body’s natural circadian rhythm, whereby glucose levels and insulin sensitivity peak in the morning and decline later in the day [4]. However, mistimed light exposure and eating from shift work disrupts this rhythm, leading to circadian misalignment and impaired glucose metabolism. Animal studies indicate that nighttime light exposure increases sympathetic activity of autonomic nerves [70,71], leading to increased gluconeogenesis, glycogenolysis, and decreased insulin release from beta cells [72]. It is postulated that a similar mechanism also occurs in humans [73], as circadian misalignment from mistimed light exposure raises glucose levels and impairs glucose tolerance [5,74]. These effects are closely tied to the SCN and clock genes, which play a pivotal role in regulating glucose metabolism [75,76].

Mistimed eating further exacerbates circadian misalignment by disrupting peripheral clocks in liver and muscle cells. Animal studies have demonstrated that feeding at unnatural times causes a phase shift in liver clocks and completely abolishes clock gene rhythms in muscle cells [77]. Aligning these clocks with feeding rhythms, rather than fixing them independently, has been found to significantly enhance glucose tolerance and regulation [78]. These findings suggest a potential mechanism whereby aligning food intake and light exposure with the body’s natural circadian rhythm could optimize and enhance glucose metabolism.

This mechanism may also explain the benefits of early TRE, which starts the eating window in the morning, aligning with the body’s natural circadian rhythm. Conversely, late TRE, starting in the afternoon or evening, misaligns with this rhythm, offering fewer benefits for glucose metabolism [79]. Early TRE has been shown to improve FBG [31,33,35] and HOMA-IR levels [33]. Additionally, two weeks of early TRE improved whole-body insulin sensitivity, reducing postprandial glucose and insulin in healthy men [80], and improved FBG, fasting blood insulin, and HOMA-IR in overweight adults [81]. While this review did not compare early- and late-TRE protocols, future studies should explore these effects in shift workers and across different demographics.

### 4.3. Sleep (Total Sleep Time, Sleep Efficiency)

Our analysis on the effect of TRE on total sleep time and sleep efficiency revealed non-significant results with negligible effect sizes, consistent with current literature suggesting minimal to negligible effects on these outcomes [82,83,84]. However, one study reported significant improvements in sleep efficiency following an 8 h TRE protocol in overweight/obese men, possibly due to different participant demographics [85]. Although our study did not analyze sleep quality due to an insufficient number of studies, a recent systematic review found that TRE could improve perceived sleep quality in shift workers [86]. These findings highlight the need for further research, to better understand the interaction between TRE and sleep in shift workers.

Notably, insufficient sleep has been correlated with multiple CMDs [87,88], with circadian misalignment serving as a key underlying mechanism [73,89]. Despite this relationship, sleep is often reported as a secondary outcome rather than a mediator in most TRE studies. A recent study examining CVD outcomes has also proposed that sleep should be considered as a mediator in the relationship between TRE and cardiovascular health [9], underscoring its potential relevance within the broader context of CMD. Therefore, future research should investigate the role of sleep alongside TRE, and its impact on CMD.

### 4.4. Implications for Future Research and Practice

Given the overall ‘very low’ certainty of evidence and non-significant findings, the effectiveness of TRE in shift workers’ glucose metabolism remains inconclusive, and findings should be interpreted with caution. However, observed FBG trends and findings from previous studies—despite inconsistencies and heterogeneity—suggest that TRE could improve glucose metabolism. Additionally, emerging evidence suggests that nighttime fasting could improve CVD risk factors under simulated shift work conditions [90], further highlighting the potential broader benefits of aligning eating patterns with the circadian rhythm for glucose metabolism and overall cardiometabolic health. This underscores the need for further research to validate these findings in shift workers.

Importantly, as the studies included in this review predominantly focused on nighttime fasting rather than TRE protocols, more rigorous studies are needed to specifically evaluate TRE in shift workers using clearly defined and consistent eating windows. This would help distinguish the effects of TRE from other forms of intermittent fasting, address the current knowledge gap regarding its efficacy, and provide more conclusive evidence to guide future research and practice.

Additionally, future TRE research in shift workers should examine additional CMD markers, such as lipid profiles, and include biomarkers of circadian rhythms, such as melatonin and cortisol, to better understand the underlying mechanisms. Given that TRE typically involves fasting for more than 12 h, future studies should meet this minimum criterion to elicit the metabolic switch for cardiometabolic benefits [61]. To reduce heterogeneity, shift schedules and participant demographics should be standardized. Furthermore, identifying the optimal TRE protocol, including the timing and duration of eating windows, and comparing early TRE with late TRE on CMD outcomes could provide insights into improving shift workers’ cardiometabolic health.

An often-overlooked factor in most TRE studies is meal composition, which could significantly influence metabolic outcomes. Meals varying in carbohydrates, fats, or protein content may elicit different metabolic responses. The lack of standardization in dietary composition and reporting across studies makes it challenging to disentangle the effects of TRE from the influence of meal composition, introducing it as a confounder. Therefore, more well-controlled studies with standardized dietary composition are necessary to better evaluate the metabolic impact of TRE in shift workers. Moreover, adherence to meal protocols was not consistently reported across studies. While adherence can be reasonably assumed in simulated shift work settings as meals were provided and monitored, both adherence and the method used to track it varied across free-living studies. Some studies employed digital tools such as mobile applications [54,55], while others relied on self-reported dietary recalls [53], and one study did not report adherence at all [56] (Supplementary Table S6). The use of subjective methods, such as self-reports, may have introduced both human error and reporting bias. Even when digital tools were used, reliance on self-reporting input limits objectivity. These differences in tracking methods could have contributed to significant heterogeneity and inconclusive findings. This highlights the need for more precise and standardized tracking methods, such as the use of digital devices or applications that minimize or eliminate self-reporting, and for consistent reporting of adherence in free-living studies in future research to enhance the reliability and comparability of findings.

While the optimal TRE protocol remains undefined, current evidence suggests that an eating window of 6–10 h—corresponding to a fasting duration of over 12 h—could improve metabolic parameters [35,91]. Furthermore, early TRE has been shown to improve glucose regulation [33], and may enhance sleep quality and efficiency, with sleep potentially acting as a mediator [85,86]. Hence, a TRE protocol starting early in the morning, with a 6–10 h eating window and proper sleep hygiene, may provide the greatest benefits for shift workers, although further research is needed.

### 4.5. Strengths and Limitations

A key strength of this review is its novelty as, to the best of our knowledge, the first to comprehensively evaluate TRE’s effectiveness on shift workers’ glucose metabolism. Additionally, the rigorous methodology, including a comprehensive search strategy incorporating gray literature, RoB-1 for methodological quality appraisal, and GRADE for overall evidence certainty, minimized publication bias and evaluated the quality of included studies. Sensitivity analyses further assessed result robustness.

However, several limitations remain. First, small sample sizes in the included studies and this review likely reduced the statistical power to detect significant effects. Second, subgroup and funnel plot analyses could not be conducted due to the small number of studies, limiting assessments of heterogeneity and publication bias. Third, all studies were conducted in Western contexts, and only English-language studies were included, limiting generalizability. Fourth, data extraction was performed by a single reviewer due to logistical constraints, introducing potential biases. Fifth, fasting durations in some studies may have been too short (<12 h) to trigger the metabolic switch, potentially reducing the physiological significance of the outcomes, limiting result reliability and data pooling. Sixth, variability in shift schedules, participant demographics, shift work settings, adherence to meal protocols, and circadian misalignment protocols introduced heterogeneity, limiting data pooling and conclusive results. Seventh, unregulated food intake in free-living shift workers caused variability and confounding effects, limiting our ability to isolate the effects of TRE from those related to the influence of meal composition. Eighth, five out of the six included studies focused on nighttime fasting rather than TRE, making it difficult to evaluate the effects of TRE on shift workers’ glucose metabolism, and contributing to the overall inconclusiveness of the findings. Ninth, the fasting and feeding windows of the nighttime fasting protocols were not explicitly stated and had to be inferred from the described protocols, which may have introduced potential biases and limited the accuracy of this review. Lastly, the overall ‘very low’ evidence certainty and inconclusive findings may have reduced credibility, validity, and certainty of findings, underscoring the need for more rigorous studies in this area.

## 5. Conclusions

This systematic review and meta-analysis included six RCTs with 316 participants to evaluate the effectiveness of TRE on shift workers’ glucose metabolism. While TRE showed non-significant trends for FBG improvement, other outcomes remained inconclusive. These findings should be interpreted with caution, given small sample sizes, overall ‘very low’ certainty of evidence, and previously identified limitations. Notably, most included studies implemented nighttime fasting protocols rather than TRE, limiting the ability to draw conclusive findings on the effect of TRE on shift workers’ glucose metabolism. Future research should examine circadian markers, dietary composition and sleep outcomes to better understand TRE’s potential benefits and mechanism. Larger, more rigorous RCTs that evaluate TRE and focus on shift workers—who are highly susceptible to circadian misalignment and impaired glucose metabolism yet are underrepresented in current literature—are essential to determine TRE’s role and efficacy in improving glucose metabolism and overall cardiometabolic health.

## Figures and Tables

**Table 1:** Characteristics of included studies.

Author, Year	Country	Design	Study Duration	ITT/Per Protocol Analysis	Population Description	Shift Work Setting	*n*	Age (Mean ± SD)	BMI (Mean ± SD)	Sex (Male: Female)	Fasting: Feeding Hours	Fasting Blood Glucose (mmol/L, Mean ± SD)	Fasting Blood Insulin (pmol/L, Mean ± SD)	HOMA-IR (Mean ± SD)	2 h Postprandial Glucose (mmol/L, Mean ± SD)
Centofanti et al., 2025 [51]	New Zealand	Parallel-group RCT 3-arm	6 days	Not stated	Healthy non-shift working adults	Simulated	55	24.5 ± 4.9 ^2^	24.0 ± 2.5 ^2^	32:23	11.5:12.5 ^6^	Fasting at night: 5.04 ± 1.098	Fasting at night: 172.2 ± 118	Fasting at night: 4.27 ± 4.12	UN
												Eating at night: 5.34 ± 1.083	Eating at night: 113.1 ± 104.4	Eating at night: 3.9 ± 3.34	
Chellappa et al., 2021 [52]	USA	Parallel-group RCT 2-arm	14 days	Per Protocol	Healthy young adults	Simulated	20	26.5 ± 4.1	22.7 ± 2.1	13:7	9.4:14.6 ^4,6^	UN	UN	UN	Fasting at night: 4.99 ± 0.918 ^5^
															Eating at night: 6.817 ± 1.053038 ^5^
Leung et al., 2021 [53]	Australia	Crossover pilot RCT 2-arm	11 weeks	Per Protocol	Shift workers with abdominal obesity	Free living	28	41 ± 10	30.7 ± 5.7	6:13 ^3^	5:19 ^6^	Fasting at night: 5.8 ± 0.6	Fasting at night: 99.6 ± 58.8 ^5^	Fasting at night: 4.4 ± 3.3	Fasting at night: 5.6 ± 1.2
												Eating at night: 5.8 + 0.5	Eating at night: 90 ± 43.2 ^5^	Eating at night: 3.9 ± 1.9	Eating at night: 5.4 ± 1.4
Manoogian et al., 2022 [54]	USA	Parallel-group RCT 2-arm	14 weeks	ITT	Firefighters with and without CVD risk factors	Free living	150	40.36 ± 9.0	Not stated	125:25	14:10	Fasting at night: 5.14 ± 0.377451 ^5^	Fasting at night: 29.04 ± 14.343136 ^5^	Fasting at night: 1.12 ± 0.566176 ^5^	UN
												Eating at night: 5.15 ± 0.389473 ^5^	Eating at night: 29.1 ± 17.956769 ^5^	Eating at night: 1.13 ± 0.737949 ^5^	
Suyoto et al., 2024 [55]	The Netherlands	Crossover RCT 2-arm	23 days	Per Protocol	Healthy nurses	Free living	53	32.5 ^1,2^	25.5 ^1,2^	0:53	10:14 ^6^	UN	UN	UN	Fasting at night: 5.24684 ± 0.726323 ^5^
															Eating at night: 5.621567 ± 0.987286 ^5^
Teixeira et al., 2023 [56]	Brazil	Crossover RCT 3-arm	28 days	Not stated	Healthy police officers	Free living	10	38.8 ± 4.0	25.9 ± 1.9	10:0	7.5:16.5 ^6^	Fasting at night: 4.84 ± 0.420583 ^5^	Fasting at night: 50.4 ± 15.811388 ^5^	Fasting at night: 1.8 ± 0.632456 ^5^	UN
												Eating at night: 4.86 ± 0.404772 ^5^	Eating at night: 119.4 ± 41.10961 ^5^	Eating at night: 4.3 ± 1.897367 ^5^	

Abbreviations: RCT (Randomized Controlled Trial), SD (Standard Deviation), ITT (Intention-To-Treat), CVD (Cardiovascular Disease), HOMA-IR (Homeostatic Model Assessment for Insulin Resistance), UN (Unknown). ^1^ SD not reported. ^2^ Weighted mean and/or pooled SD calculated. ^3^ Calculated after accounting for attrition. ^4^ Converted from a 28 h circadian misalignment protocol where fasting: feeding hours is 11:17. ^5^ Calculated after converting units and/or converting SE and 95% CI to SD, where necessary. ^6^ Nighttime fasting protocols.

## Supplementary Materials

The following supporting information can be downloaded at: https://www.mdpi.com/article/10.3390/nu17101689/s1, Table S1: PRISMA checklist; Table S2: Eligibility criteria; Table S3: Search strategy with results; Table S4: Extracted means and SE/SEM from graphical representation using WebPlotDigitizer; Table S5: Unit conversion [92]; Table S6: Characteristics of included studies (detailed); Table S7: GRADE assessment of evidence certainty; Table S8: Emails sent to corresponding researchers on missing data or typological errors; Figure S1: Email chain between Dr. Crispim and J.Y.J.K. regarding correction of reported insulin unit error.

## Abbreviations

The following abbreviations are used in this manuscript:

BMI	Body mass index
FBG	Fasting blood glucose
CMD	Cardiometabolic disease
SCN	Suprachiasmatic nucleus
TRE	Time-restricted eating
HbA1c	Glycated hemoglobin
PRISMA	Preferred Reporting Items for Systematic Reviews and Meta-Analyses
PROSPERO	International Prospective Register of Systematic Reviews
PICO	Population, intervention, control, outcome
HOMA-IR	Homeostatic Model Assessment of Insulin Resistance
RCT	Randomized controlled trial
SD	Standard deviation
RoB-1	Risk of bias-1
GRADE	Grading of Recommendations, Assessment, Development, and Evaluation
CI	Confidence interval
SE	Standard error
WMD	Weighted mean difference
SMD	Standardized mean difference
CVD	Cardiovascular disease
ITT	Intention-to-treat
