# Supplementary material for: The Effectiveness of Time-Restricted Eating as an Intermittent Fasting Approach on Shift Workers’ Glucose Metabolism: A Systematic Review and Meta-Analysis
Source: Nutrients. 2025 May 15;17(10):1689. doi: 10.3390/nu17101689 (PMC12114545; doi:10.3390/nu17101689)
Supplement: Supplementary file 1 [file nutrients-17-01689-s001.zip › nutrients-3616576-supplementary.pdf]

# The Effectiveness of Time-Restricted Eating on Shift Workers' Glucose Metabolism: A Systematic Review and Meta-Analysis

## Supplementary Data

**Supplemental Table S1.** PRISMA Checklist.

| Section and Topic | Item # | Checklist item | Location where item is reported |
|---|---|---|---|
| **TITLE** | | | |
| Title | 1 | Identify the report as a systematic review. | 1 |
| **ABSTRACT** | | | |
| Abstract | 2 | See the PRISMA 2020 for Abstracts checklist. | 1 |
| **INTRODUCTION** | | | |
| Rationale | 3 | Describe the rationale for the review in the context of existing knowledge. | 2-3 |
| Objectives | 4 | Provide an explicit statement of the objective(s) or question(s) the review addresses. | 2-3 |
| **METHODS** | | | |
| Eligibility criteria | 5 | Specify the inclusion and exclusion criteria for the review and how studies were grouped for the syntheses. | 3 |
| Information sources | 6 | Specify all databases, registers, websites, organisations, reference lists and other sources searched or consulted to identify studies. Specify the date when each source was last searched or consulted. | 3-4 |
| Search strategy | 7 | Present the full search strategies for all databases, registers and websites, including any filters and limits used. | 3-4 |
| Selection process | 8 | Specify the methods used to decide whether a study met the inclusion criteria of the review, including how many reviewers screened each record and each report retrieved, whether they worked independently, and if applicable, details of automation tools used in the process. | 3-4 |
| Data collection process | 9 | Specify the methods used to collect data from reports, including how many reviewers collected data from each report, whether they worked independently, any processes for obtaining or confirming data from study investigators, and if applicable, details of automation tools used in the process. | 4 |
| Data items | 10a | List and define all outcomes for which data were sought. Specify whether all results that were compatible with each outcome domain in each study were sought (e.g. for all measures, time points, analyses), and if not, the methods used to decide which results to collect. | 3-4 |
| | 10b | List and define all other variables for which data were sought (e.g. participant and intervention characteristics, funding sources). Describe any assumptions made about any missing or unclear information. | 3-4 |
| Study risk of bias assessment | 11 | Specify the methods used to assess risk of bias in the included studies, including details of the tool(s) used, how many reviewers assessed each study and whether they worked independently, and if applicable, details of automation tools used in the process. | 4 |
| Effect measures | 12 | Specify for each outcome the effect measure(s) (e.g. risk ratio, mean difference) used in the synthesis or presentation of results. | 5 |
| Synthesis methods | 13a | Describe the processes used to decide which studies were eligible for each synthesis (e.g. tabulating the study intervention characteristics and comparing against the planned groups for each synthesis (item #5)). | 3-4 |
| | 13b | Describe any methods required to prepare the data for presentation or synthesis, such as handling of missing summary statistics, or data conversions. | 4 |

| Section and Topic | Item # | Checklist item | Location where item is reported |
|---|---|---|---|
| | 13c | Describe any methods used to tabulate or visually display results of individual studies and syntheses. | 5 |
| | 13d | Describe any methods used to synthesize results and provide a rationale for the choice(s). If meta-analysis was performed, describe the model(s), method(s) to identify the presence and extent of statistical heterogeneity, and software package(s) used. | 5 |
| | 13e | Describe any methods used to explore possible causes of heterogeneity among study results (e.g. subgroup analysis, meta-regression). | 5 |
| | 13f | Describe any sensitivity analyses conducted to assess robustness of the synthesized results. | 5 |
| Reporting bias assessment | 14 | Describe any methods used to assess risk of bias due to missing results in a synthesis (arising from reporting biases). | 4 |
| Certainty assessment | 15 | Describe any methods used to assess certainty (or confidence) in the body of evidence for an outcome. | 4-5 |
| **RESULTS** | | | |
| Study selection | 16a | Describe the results of the search and selection process, from the number of records identified in the search to the number of studies included in the review, ideally using a flow diagram. | 5-6 |
| | 16b | Cite studies that might appear to meet the inclusion criteria, but which were excluded, and explain why they were excluded. | 5-6 |
| Study characteristics | 17 | Cite each included study and present its characteristics. | 6-7 |
| Risk of bias in studies | 18 | Present assessments of risk of bias for each included study. | 8-9 |
| Results of individual studies | 19 | For all outcomes, present, for each study: (a) summary statistics for each group (where appropriate) and (b) an effect estimate and its precision (e.g. confidence/credible interval), ideally using structured tables or plots. | 9-12 |
| Results of syntheses | 20a | For each synthesis, briefly summarise the characteristics and risk of bias among contributing studies. | 6-9 |
| | 20b | Present results of all statistical syntheses conducted. If meta-analysis was done, present for each the summary estimate and its precision (e.g. confidence/credible interval) and measures of statistical heterogeneity. If comparing groups, describe the direction of the effect. | 9-12 |
| | 20c | Present results of all investigations of possible causes of heterogeneity among study results. | 12 |
| | 20d | Present results of all sensitivity analyses conducted to assess the robustness of the synthesized results. | 12 |
| Reporting biases | 21 | Present assessments of risk of bias due to missing results (arising from reporting biases) for each synthesis assessed. | 8-9 |
| Certainty of evidence | 22 | Present assessments of certainty (or confidence) in the body of evidence for each outcome assessed. | 8 |
| **DISCUSSION** | | | |
| Discussion | 23a | Provide a general interpretation of the results in the context of other evidence. | 12-14 |

| Section and Topic | Item # | Checklist item | Location where item is reported |
|---|---|---|---|
| | 23b | Discuss any limitations of the evidence included in the review. | 16 |
| | 23c | Discuss any limitations of the review processes used. | 16 |
| | 23d | Discuss implications of the results for practice, policy, and future research. | 14-15 |
| **OTHER INFORMATION** | | | |
| Registration and protocol | 24a | Provide registration information for the review, including register name and registration number, or state that the review was not registered. | 3 |
| | 24b | Indicate where the review protocol can be accessed, or state that a protocol was not prepared. | 3 |
| | 24c | Describe and explain any amendments to information provided at registration or in the protocol. | NA |
| Support | 25 | Describe sources of financial or non-financial support for the review, and the role of the funders or sponsors in the review. | NA |
| Competing interests | 26 | Declare any competing interests of review authors. | NA |
| Availability of data, code and other materials | 27 | Report which of the following are publicly available and where they can be found: template data collection forms; data extracted from included studies; data used for all analyses; analytic code; any other materials used in the review. | NA |

**Supplemental Table S2.** Eligibility Criteria.

| Criteria | Inclusion criteria | Exclusion criteria |
|---|---|---|
| **Population** | - Shift workers (free-living)<br>- Individuals who are involved in a lab-based shift work simulation study | - Shift workers who do not participate in night shifts<br>- Animals<br>- Individuals with acute or chronic diseases, such as diabetes or hypertension |
| **Intervention** | - TRE, only fasting during the night shift/overnight, regardless of the duration of the eating window | - Fasting during the alternative day<br>- Ramadan fasting<br>- Other interventions in conjunction with TRE |
| **Comparison** | - Able to eat during the night shift/overnight | NA |
| **Primary outcome** | - Plasma glucose<br>- Plasma insulin<br>- HOMA-IR<br>- 2h-postprandial glucose | |
| **Secondary outcomes** | - Total sleep time<br>- Sleep efficiency | - Fasting Triglycerides<br>- Fasting Total Cholesterol<br>- Fasting HDL-c<br>- Fasting LDL-c<br>- Postprandial glucose (AUC)<br>- Postprandial glucose (peak)<br>- Postprandial insulin (peak)<br>- Postprandial insulin (AUC)<br>- Postprandial NEFA<br>Postprandial TAG<br>- Postprandial pancreatic polypeptide<br>- Postprandial glucagon<br>- Energy expenditure<br>- Activity<br>- Body temperature<br>- Plasma Cortisol<br>- Body weight<br>- BMI<br>- Body Fat<br>- CRP<br>- HbA1c<br>- BP<br>- TSH<br>- T3, T4 hormones |
| **Type of design** | - RCTs | - Non-RCTs<br>- Secondary research articles |
| **Year of publication** | No limit | NA |
| **Publication type** | - Articles from Internationally Refereed Journals<br>- Grey literature – theses, full text conference papers | - Protocols<br>- Abstract only<br>- Summaries<br>- Expert commentaries<br>- Books |
| **Language** | - English language | - A language other than English language |

**Supplemental Table S3.** Search Strategy with Results.

| | | | |
|---|---|---|---|
| Clean Version | P | "Shift work*" OR Shiftwork OR "night work" OR "night shift*" OR "Night duty*" OR "night schedule*" OR "alternating schedule*" | |
| | I | ("time restrict*" OR intermittent) AND (eating* OR feed* OR fast* OR diet* OR meal* OR "energy restrict*") | |
| | O | insulin OR glucose OR metabolic OR "postprandial hormone" OR "preprandial hormone" | |
| PubMed 24/9/24 | P | #1: "Shift work*"[Title/Abstract] OR Shiftwork[Title/Abstract] OR "night work"[Title/Abstract] OR "night shift*"[Title/Abstract] OR "Night duty*"[Title/Abstract] OR "night schedule*"[Title/Abstract] OR "alternating schedule*"[Title/Abstract] | 10548 |
| | | #2: "Shift Work Schedule"[Mesh] OR "Work Schedule Tolerance"[Mesh] | 8491 |
| | | #3: #1 OR #2 | 15188 |
| | I | #4: ("time restrict*"[Title/Abstract] OR intermittent[Title/Abstract]) AND (eating*[Title/Abstract] OR feed*[Title/Abstract] OR fast*[Title/Abstract] OR diet*[Title/Abstract] OR meal*[Title/Abstract] OR "energy restrict*"[Title/Abstract]) | 9714 |
| | | #5: "Fasting"[Mesh] OR "Feeding Behavior"[Mesh] | 199253 |
| | | #6: #4 OR #5 | 207140 |
| | O | #7: insulin[Title/Abstract] OR glucose[Title/Abstract] OR metabolic[Title/Abstract] OR "postprandial hormone"[Title/Abstract] OR "preprandial hormone"[Title/Abstract] | 1380681 |
| | | #8: "Glucose"[Mesh] OR "Insulin"[Mesh] | 45729 |
| | | #9: #7 OR #8 | 1470711 |
| | | #10: #3 AND #6 AND #9 | 135 |
| EMBASE 24/9/24 | P | #1: 'shift work'/exp OR 'shift worker'/exp | 12850 |
| | | #2: 'shift work*':ab,ti OR shiftwork:ab,ti OR 'night work':ab,ti OR 'night shift*':ab,ti OR 'night duty*':ab,ti OR 'night schedule*':ab,ti OR 'alternating schedule*':ab,ti | 14801 |
| | | #3: #1 OR #2 | 19153 |
| | I | #4: ‘feeding behaviour’/exp OR ‘diet restriction’/exp OR ‘meal skipping’/exp | 387191 |
| | | #5: ('time restrict*':ab,ti OR intermittent:ab,ti) AND (eating*:ab,ti OR feed*:ab,ti OR fast*:ab,ti OR diet*:ab,ti OR meal*:ab,ti OR 'energy restrict*':ab,ti) | 14307 |
| | | #6: #4 OR #5 | 397339 |
| | O | #7: glucose/exp OR insulin/exp | 818300 |
| | | #8: insulin:ab,ti OR glucose:ab,ti OR metabolic:ab,ti OR 'postprandial hormone':ab,ti OR 'preprandial hormone':ab,ti | 1807468 |
| | | #9: #7 OR #8 | 2003640 |
| | | #10: #3 AND #6 AND #9 | 366 |
| The Cochrane Library (trials only) 24/9/24 | P | #1: ((Shift NEXT work*) OR Shiftwork OR "night work" OR (night NEXT shift*) OR (Night NEXT duty*) OR (night NEXT schedule*) OR (alternating NEXT schedule*)):ti,ab | 1074 |
| | | #2: MeSH descriptor: [Shift Work Schedule] explode all trees | 56 |
| | | #3: MeSH descriptor: [Work Schedule Tolerance] explode all trees | 237 |
| | | #4: #1 OR #2 OR #3 | 1167 |
| | I | #5: ((time NEXT restrict*) OR intermittent) AND (eating* OR feed* OR fast* OR diet* OR meal* OR (energy NEXT restrict*)):ti,ab | 2732 |
| | | #6: MeSH descriptor: [Fasting] explode all trees | 4499 |
| | | #7: MeSH descriptor: [Feeding Behavior] explode all trees | 12981 |
| | | #8: #5 OR #6 OR #7 | 15482 |
| | O | #9: (insulin OR glucose OR metabolic OR "postprandial hormone" OR "preprandial hormone"):ti,ab | 125428 |
| | | #10: MeSH descriptor: [Glucose] explode all trees | 25821 |
| | | #11: MeSH descriptor: [Insulins] in all MeSH products | 18739 |
| | | #12: #9 OR #10 OR #11 | 129097 |

| | | | |
|---|---|---|---|
| | | #13: #4 AND #8 AND #12 | 18 |
| CINAHL 24/9/24 | P | #1: (MM "Shiftwork") OR (MM "Shift Workers") | 3173 |
| | | #2: TI ( "Shift work*" OR Shiftwork OR "night work" OR "night shift*" OR "Night duty*" OR "night schedule*" OR "alternating schedule*" ) OR AB ( "Shift work*" OR Shiftwork OR "night work" OR "night shift*" OR "Night duty*" OR "night schedule*" OR "alternating schedule*" ) | 4335 |
| | | #3: #1 OR #2 | 5944 |
| | I | #4: (MH "Eating Behavior+") | 53243 |
| | | #5: TI ( ("time restrict*" OR intermittent) AND (eating* OR feed* OR fast* OR diet* OR meal* OR "energy restrict*") ) OR AB ( ("time restrict*" OR intermittent) AND (eating* OR feed* OR fast* OR diet* OR meal* OR "energy restrict*") ) | 2195 |
| | | #6: #4 OR #5 | 54724 |
| | O | #7: (MM "Glucose") OR (MH "Insulin+") OR (MM "Blood Glucose") | 50123 |
| | | #8: TI ( insulin OR glucose OR metabolic OR "postprandial hormone" OR "preprandial hormone" ) OR AB ( insulin OR glucose OR metabolic OR "postprandial hormone" OR "preprandial hormone" ) | 185997 |
| | | #9: #7 OR #8 | 196588 |
| | | #10: #3 AND #6 AND #9 | 40 |
| PsycINFO 24/9/24 | P | #1: ("Shift work*" or Shiftwork or "night work" or "night shift*" or "Night duty*" or "night schedule*" or "alternating schedule*").ab,ti. | 3411 |
| | | #2: exp workday shifts/ | 2795 |
| | | #3: exp work scheduling/ | 2105 |
| | | #4: #1 OR #2 OR #3 | 6140 |
| | I | #5: ("time restrict*" or intermittent).mp. and (eating* or feed* or fast* or diet* or meal* or "energy restrict*").ab,ti. | 1198 |
| | | #6: exp eating behavior/ | 28789 |
| | | #7: #5 OR #6 | 29854 |
| | O | #8: (insulin or glucose or metabolic or "postprandial hormone" or "preprandial hormone").ab,ti. | 50115 |
| | | #9: exp glucose/ | 5715 |
| | | #10: exp insulin/ | 4357 |
| | | #11: #8 OR #9 OR #10 | 50780 |
| | | #12: #4 AND #7 AND #11 | 14 |
| Scopus 24/9/24 | P | #1: TITLE-ABS-KEY ( "Shift work*" OR shiftwork OR "night work" OR "night shift*" OR "Night duty*" OR "night schedule*" OR "alternating schedule*" ) | 18563 |
| | I | #2: TITLE-ABS-KEY ( ( "time restrict*" OR intermittent ) AND ( eating* OR feed* OR fast* OR diet* OR meal* OR "energy restrict*" ) ) | 22447 |
| | O | #3: TITLE-ABS-KEY ( insulin OR glucose OR metabolic OR "postprandial hormone" OR "preprandial hormone" ) | 2408177 |
| | | #4: #1 AND #2 AND #3 | 60 |
| Web of Science 24/9/24 | P | #1: (TI=("Shift work*" OR Shiftwork OR "night work" OR "night shift*" OR "Night duty*" OR "night schedule*" OR "alternating schedule*")) OR AB=("Shift work*" OR Shiftwork OR "night work" OR "night shift*" OR "Night duty*" OR "night schedule*" OR "alternating schedule*") | 11503 |
| | I | #2: (TI=(("time restrict*" OR intermittent) AND (eating* OR feed* OR fast* OR diet* OR meal* OR "energy restrict*"))) OR AB=(("time restrict*" OR intermittent) AND (eating* OR feed* OR fast* OR diet* OR meal* OR "energy restrict*")) | 14975 |
| | O | #3: (TI=(insulin OR glucose OR metabolic OR "postprandial hormone" OR "preprandial hormone")) OR AB=(insulin OR glucose OR metabolic OR "postprandial hormone" OR "preprandial hormone") | 1649005 |
| | | #4: #1 AND #2 AND #3 | 26 |

| | | | |
|---|---|---|---|
| ProQuest Dissertations and Theses 24/9/24 | P | #1: title("Shift work*" OR Shiftwork OR "night work" OR "night shift*" OR "Night duty*" OR "night schedule*" OR "alternating schedule*") OR abstract("Shift work*" OR Shiftwork OR "night work" OR "night shift*" OR "Night duty*" OR "night schedule*" OR "alternating schedule*") | 1144 |
| | I | #2: title(("time restrict*" OR intermittent) AND (eating* OR feed* OR fast* OR diet* OR meal* OR "energy restrict*")) OR abstract(("time restrict*" OR intermittent) AND (eating* OR feed* OR fast* OR diet* OR meal* OR "energy restrict*")) | 1557 |
| | O | #3: title(insulin OR glucose OR metabolic OR "postprandial hormone" OR "preprandial hormone") OR abstract(insulin OR glucose OR metabolic OR "postprandial hormone" OR "preprandial hormone") | 91035 |
| | | #4: #1 AND #2 AND #3 | 2 |
| Science.gov 24/9/24 | | ("Shift work*" OR Shiftwork OR "night work" OR "night shift*" OR "Night duty*" OR "night schedule*" OR "alternating schedule*")<br>AND<br>(("time restrict*" OR intermittent) AND (eating* OR feed* OR fast* OR diet* OR meal* OR "energy restrict*")) AND<br>(insulin OR glucose OR metabolic OR "postprandial hormone" OR "preprandial hormone") | 216 |
| Clinicaltrials.gov 24/9/24 | | Condition/disease: Glucose Metabolism<br>Other terms: Insulin Sensitivity<br>Intervention/treatment: Time Restricted Eating | 25 |

**Supplemental Table S4.** Extracted Means and SE/SEM from Graphical Representations using *WebPlotDigitizer*

| Author, Year | Outcome | Study Group | Mean | [1]Lower Bound SEM/SE | [1]Upper Bound SEM/SE |
|---|---|---|---|---|---|
| Chellappa et al., 2021 | 2h Postprandial Glucose (mg/dL) | Fasting at night | 89.80 | 84.24 | 95.34 |
| | | Eating at night | 122.71 | 116.02 | 128.64 |
| Suyoto et al., 2024 | 2h Postprandial Glucose (mmol/L) | Fasting at night | 5.25 | 5.17 | 5.32 |
| | | 1-meal at night | 5.62 | 5.51 | 5.71 |

*[1]SEM values are reported for Chellappa et al. (2021) study, while SE values are reported for Suyoto et al. (2024) study*

**Supplemental Table S5.** Unit Conversion.

| Units given | Units converted to | Conversion Factor |
|---|---|---|
| mg/dL | mmol/L | 1mg/dL = 0.0555mmol/L (divide by 18) |
| µU/mL | pmol/L | 1µU/mL = 6 pmol/L [87] |
| mU/mL | pmol/L | 1µU/mL = 6 pmol/L [87] |
| mIU/L | pmol/L | 1mIU/L = 6 pmol/L [87] |
| Hours | Minutes | 1 hour = 60 minutes |

**Supplemental Table S6.** Characteristics of Included Studies (Detailed).

| Author, Year | Country | Design | Study Duration | ITT/per protocol analysis | Population Description | Shift Work Setting | Total Number Randomised (n=) | Withdrawals and Exclusions (n=) | Age (mean ± SD) | | BMI (mean ± SD) | | Sex (no. of males:females) | Duration of Treatment | Fasting: Feeding hours | Energy Intake Distribution and Time of Meal | Adherence to Meals | Fasting Blood Glucose (mmol/L, mean± SD) | Fasting Blood Insulin (pmol/L, mean± SD) | HOMA-IR (mean± SD) | 2-hour Postprandial Glucose (mmol/L, mean± SD) | Total Sleep Time (mins, mean± SD) | Sleep Efficiency (%, mean± SD) |
|---|---|---|---|---|---|---|---|---|---|---|---|---|---|---|---|---|---|---|---|---|---|---|---|
| **Centofani et al., 2024** | New Zealand | Parallel-group RCT 3-arm | 6 days | Not stated | Healthy non-shift working adults | Simulated | Fasting at night: 20 | 3 | Fasting at night: 24.7 ± 5.4 | Weighted mean and pooled SD: 24.5 ± 4.9 | Fasting at night: 23.9 ± 2.3 | Weighted mean and pooled SD: 24.0 ± 2.5 | 32:23 | 4 days | 11.5:12.5[7] | Fasting at night: 40% (1900), 30% (0630), 10% (0930), 20% (1700) | Participants encouraged to finish provided meals. | Fasting at night: 5.04±1.098 | Fasting at night: 172.2±118 | Fasting at night: 4.27±4.12 | UN | Fasting at night: 348±66[6] | Fasting at night: 82.5±15.7 |
| | | | | | | | Snack at night: 17 | | Snacking at night: 25.4 ± 5.6 | | Snacking at night: 23.6 ± 1.9 | | | | | Snacking at night: 40% (1900), 10% (0030), 30% (0630), 20% (1700) | | NA | NA | NA | NA | NA | NA |
| | | | | | | | Meal at night: 18 | | Meal at night: 23.5 ± 3.5 | | Meal at night: 24.6 ± 3.2 | | | | | Meal at night: 40% (1900), 30% (0030), 30% (0630) | | Meal at night: 5.34±1.083 | Meal at night: 113.1±104.4 | Meal at night: 3.9±3.34 | UN | Meal at night: 360±66[6] | Meal at night: 88.8±12.9 |

| | | | | | | | | | | | | | | | | | | | | | | | |
|---|---|---|---|---|---|---|---|---|---|---|---|---|---|---|---|---|---|---|---|---|---|---|---|
| **Chellappa et al., 2021** | USA | Parallel-group RCT 2-arm | 14 days | per protocol | Healthy young adults | Simulated | Fasting at night: 10 | 1 | 26.5 ± 4.1 | | 22.7 ± 2.1 | | 13:7 | 4 days | 9.4:14.6[5,7] | Breakfast - 33.3%, Lunch - 23.4%, Snack - 10%, Dinner - 33.3%[2] | Participants instructed to consume provided meals, meal trays checked. | UN | UN | UN | Fasting at night: 4.99±0.918[6] | UN | UN |
| | | | | | | | Eating at night: 10 | | | | | | | | | | | UN | UN | UN | Eating at night: 6.817±1.053038[6] | UN | UN |
| **Leung et al., 2021** | Australia | Crossover pilot RCT 2-arm | 11 weeks | per protocol | Night shift workers with abdominal obesity | Free-Living | 28 | 9 | 41 ± 10 | | 30.7 ± 5.7 | | 6:13[4] | 4 weeks | 5:19[7] | 0100-0600[3] | Adherence to meals verified and reported by 24-hour dietary recalls via phone calls, once weekly. | Fasting at night: 5.8±0.6 | Fasting at night: 99.6±58.8[6] | Fasting at night: 4.4±3.3 | Fasting at night: 5.6±1.2 | UN | UN |
| | | | | | | | | | | | | | | | | | | Eating at night: 5.8+0.5 | Eating at night: 90±43.2[6] | Eating at night: 3.9±1.9 | Eating at night: 5.4±1.4 | UN | UN |
| **Manoogian et al., 2022** | USA | Parallel-group RCT 2-arm | 14 weeks | ITT | Firefighters working 24h shifts (with and without CVD risk factors) | Free-Living | Fasting at night :75 | 13 | 40.36 ± 9.0 | | Not stated | | 125:25 | 12 weeks | 14:10 | Self-selected 10h eating window[3] | Adherence to meals verified and reported using myCircadianClock, a smartphone application, and 24-hour dietary recalls. | Fasting at night: 5.14±0.377451[6] | Fasting at night: 29.04±14.343136[6] | Fasting at night: 1.12±0.566176[6] | UN | Fasting at night: 478.08±49.404134[6] | Fasting at night: 90.23±2.390523[6] |
| | | | | | | | Eating at night: 75 | | | | | | | | | | | Eating at night: 5.15±0.389473[6] | Eating at night: 29.1±17.956769[6] | Eating at night: 1.13±0.737949[6] | UN | Eating at night: 471.65±45.773361[6] | Eating at night: 89.75±2.76731[6] |
| **Suyoto et al.,** | Netherlands | Crossover RCT | 23 days | per protocol | Healthy female night | Free-Living | Fasting at night: NA | 4 | Fasting at night: NA | Weighted mean: 32.5 | Fasting at night: NA | Weighted mean: 25.5 | 0:53 | 9 days | 10:14[7] | Fasting at night: 35% (1800), | Adherence to meals verified and | UN | UN | UN | Fasting at night: 5.24684 | UN | UN |

| | | | | | | | | | | | | | | | | | | | | | |
|---|---|---|---|---|---|---|---|---|---|---|---|---|---|---|---|---|---|---|---|---|---|
| **2024** | | 2-arm | | | shift nurses | | | | | | | | | 5% (2000), 30% (2200), 20% (0800), 10% (1600) | reported using Traqq, a food diary smartphone application. | | | | ±0.726323[6] | | |
| | | | | | | | 1-meal at night: 26 | | 1-meal at night: 32[1] | 1-meal at night: 26[1] | | | | 1-meal at night: 35% (1800), 5% (2000), 20% (2200), 10% (0230), 20% (0800), 10% (1600) | | UN | UN | UN | 1-meal at night: 5.621567±0.987286[6] | UN | UN |
| | | | | | | | 3-meal at night: 27 | | 3-meal at night: 33[1] | 3-meal at night: 25[1] | | | | 3-meal at night: 35% (1800), 5% (2000), 10% (0000), 10% (0230), 10% (0500), 20% (0800), 10% (1600) | | NA | NA | NA | NA | NA | NA |
| **Teixeira et al., 2022** | Brazil | Crossover RCT 2-arm | 28 days | Not stated | Healthy male night shift police officers | Free-Living | 10 | 0 | 38.8 ± 4.0 | 25.9 ± 1.9 | 10:0 | 9 days | 7.5:16.5[7] | Fasting at night: 100% consumed before 2200 | Adherence to meals collected via self-reporting and | Fasting at night: 4.84±0.420583[6] | Fasting at night: 50.4±15.811388[6] | Fasting at night: 1.8±0.632456[6] | UN | UN | UN |

| | | | | | | | | |
|---|---|---|---|---|---|---|---|---|
| | Eating at night: 70% consumed before 2200, 30% consumed after (0200) | verified with trained nutritionist. Adherence not reported. | Eating at night: 4.86±0.404772[6] | Eating at night: 119.4±41.10961[6] | Eating at night: 4.3±1.897367[6] | UN | UN | UN |
| | Sleeping at night: 100% consumed before 2200 | | NA | NA | NA | NA | NA | NA |

Abbreviations: RCT (Randomized Controlled Trial), SD (Standard Deviation), ITT (Intention-to-Treat), CVD (Cardiovascular Disease), HOMA-IR (Homeostatic Model Assessment for Insulin Resistance), UN (Unknown), NA (Not Applicable).

[1]SD not reported. Study includes 2 study groups and 3 intervention periods

[2]Time of meal not reported

[3]Energy intake distribution not reported

[4]Calculated after accounting for attrition

[5]Converted from a 28-hour circadian misalignment protocol where fasting:feeding is 11:17

[6]Calculated after converting units and /or converting SE and 95% CI to SD, where necessary.

[7]Nighttime fasting protocols.

**Supplemental Table S7.** GRADE Assessment of Evidence Certainty.

| Certainty assessment | | | | | | | № of patients | | Effect | | Certainty | Importance |
|---|---|---|---|---|---|---|---|---|---|---|---|---|
| № of studies | Study design | Risk of bias | Inconsistency | Indirectness | Imprecision | Other considerations | TRE | eating at night | Relative (95% CI) | Absolute (95% CI) | | |
| **Fasting Glucose** | | | | | | | | | | | | |
| 5 | randomised trials | serious[a] | not serious | not serious | very serious[b] | publication bias strongly suspected[c] | 125 | 117 | - | MD **0.02 mmol/L lower** (0.13 lower to 0.09 higher) | ⊕◯◯◯ Very low[a,b,c] | CRITICAL |
| **Fasting Insulin** | | | | | | | | | | | | |
| 5 | randomised trials | serious[a] | very serious[d] | not serious | very serious[b] | publication bias strongly suspected[c] | 125 | 117 | - | MD **10.17 pmol/L higher** (11.95 lower to 32.29 higher) | ⊕◯◯◯ Very low[a,b,c,d] | CRITICAL |
| **HOMA-IR** | | | | | | | | | | | | |
| 4 | randomised trials | serious[a] | very serious[d] | not serious | very serious[b] | publication bias strongly suspected[c] | 118 | 113 | - | MD **0.5 lower** (1.89 lower to 0.89 higher) | ⊕◯◯◯ Very low[a,b,c,d] | CRITICAL |
| **Postprandial Glucose** | | | | | | | | | | | | |
| 4 | randomised trials | serious[a] | very serious[d] | not serious | serious[e] | publication bias strongly suspected[c] | 59 | 58 | - | MD **0.01 mmol/L lower** (1.24 lower to 1.22 higher) | ⊕◯◯◯ Very low[a,c,d,e] | CRITICAL |
| **Postprandial Insulin** | | | | | | | | | | | | |
| 2 | randomised trials | serious[a] | not serious | not serious | very serious[b] | publication bias strongly suspected[c] | 23 | 21 | - | MD **32.48 pmol/L higher** (117.91 lower to 182.87 higher) | ⊕◯◯◯ Very low[a,b,c] | CRITICAL |

| Certainty assessment | | | | | | | № of patients | | Effect | | Certainty | Importance |
|---|---|---|---|---|---|---|---|---|---|---|---|---|
| № of studies | Study design | Risk of bias | Inconsistency | Indirectness | Imprecision | Other considerations | TRE | eating at night | Relative (95% CI) | Absolute (95% CI) | | |
| **Total Sleep Time** | | | | | | | | | | | | |
| 3 | randomised trials | serious[a] | not serious | not serious | serious[b] | publication bias strongly suspected[c] | 96 | 88 | - | SMD **0.07 SD higher** (0.22 lower to 0.36 higher) | ⊕◯◯◯ Very low[a,b,c] | IMPORTANT |
| **Sleep Efficiency** | | | | | | | | | | | | |
| 3 | randomised trials | serious[a] | not serious | not serious | serious[b] | publication bias strongly suspected[c] | 96 | 88 | - | SMD **0.01 SD lower** (0.4 lower to 0.39 higher) | ⊕◯◯◯ Very low[a,b,c] | IMPORTANT |

**CI:** confidence interval; **MD:** mean difference; **SMD:** standardised mean difference

## Explanations

a. Unclear and high risk of bias present in multiple domains.

b. small sample size and wide CI

c. unable to run funnel plot due to small sample size

d. high heterogeneity and non-overlapping CI

e. small sample size

**Supplemental Table S8.** Emails Sent to Corresponding Researchers on Missing Data or Typological Errors.

| Author, Year | Email query | Reply |
|---|---|---|
| Chellappa et al., 2021 | To request for 2-hour postprandial glucose, 2-hour postprandial insulin, and energy expenditure data[1] | Did not reply. |
| Suyoto et al., 2024 | To request for 2-hour postprandial glucose data | Did not reply. |
| Centofanti et al., 2025 | To request for FBG, fasting blood insulin, HOMA-IR, 2-hour postprandial glucose, and 2-hour postprandial insulin data[1] | Responded initially but did not provide results in follow-up email. |
| Teixeira et al., 2023 | To clarify whether the reported insulin unit was an error. | Confirmed an error in the reported insulin unit; it should be mU/mL instead of mg/dL. |

[1]2-hour postprandial insulin and energy expenditure were excluded as outcomes due to the limited number of studies available for a meta-analysis or meaningful narrative synthesis.

Outlook

## Re: Clarification on Fasting Insulin Values

**From** Cibele Crispim <cibelecrispim@gmail.com>
**Date** Fri 22/11/2024 8:55 PM
**To** Koh Jia Ying Jennell <jennell.koh.jy@u.nus.edu>; Bruno Simão Teixeira <brunosimao2005@hotmail.com>

- External Email -

Dear Koh Jia Ying Jennell,

Thank you for reaching out and for your interest in our work. I greatly appreciate your careful reading of our article and your thoughtful observation regarding the fasting insulin values.

Upon reviewing the data and the publication, you are correct that the units for fasting insulin were incorrectly reported in the tables as mg/dL. This was a typographical error. The original laboratory data, which we have re-examined, indicate that fasting insulin values were reported in **mU/mL** (as per standard laboratory practice). Additionally, the description in the "Study Protocol - Outcomes - Metabolic Parameters" section also mistakenly mirrored the unit for glucose.

We sincerely apologize for this oversight and are grateful that you brought it to our attention. Moving forward, please consider the correct unit for fasting insulin as **mU/mL** when integrating the values into your analysis.

Thank you again for your careful work and for bringing this issue to light. If you have further questions or need additional clarification, please do not hesitate to reach out.

Best regards,

Cibele Crispim
Professor, Nutrition Course, School of Medicine
Federal University of Uberlândia (FAMED-UFU)
Av. Pará, 1720, Bloco 2U, sala 20.
Campus Umuarama
Zipcode 38.405-320 Uberlândia- MG
Office number +55 34 3218-2084
http://lattes.cnpq.br/9865330615540205
https://orcid.org/0000-0002-6638-8197
ResearcherID: Cibele CAC Crispim | Publons

Em qua., 20 de nov. de 2024 às 07:35, Koh Jia Ying Jennell <e0773194@u.nus.edu> escreveu:

> Dear Dr Crispim,

I hope this email finds you well. My name is Jennell, and I am currently conducting a study that involves fasting insulin data. I came across your article, *"Influence of fasting during the night shift on next day eating behavior, hunger, and glucose and insulin levels: a randomized, three-condition, crossover trial"* (https://doi.org/10.1007/s00394-022-03069-6), and found it highly relevant to my work.

Influence of fasting during the night shift on next day eating behavior, hunger, and glucose and insulin levels: a randomized, three-condition, crossover trial - European Journal of Nutrition

Aims To investigate the influence of fasting during the night shift on eating behavior, hunger, glucose and insulin levels the following day. Methods Study with 10 male police officers who have been working at night. Participants were tested under three [illegible]

doi.org

While using the fasting insulin values reported in your paper, I converted the units from mg/dL (as stated) to pmol/L for consistency with my dataset. However, the resulting numbers seem unusually large and out of the expected range. This raised some concern about whether there might have been a reporting error or a difference in unit interpretation.

Could you kindly confirm if the fasting insulin values in your paper were reported in mg/dL or another unit? If possible, I would greatly appreciate access to the original data or clarification on the reported values to ensure accurate integration into my analysis.

Thank you for your time and for your contribution to this field of research. I look forward to your response.

Best regards,
Koh Jia Ying Jennell
Honours Student
e0773194@u.nus.edu
NUS Alice Lee Centre for Nursing Studies
National University of Singapore
21 Lower Kent Ridge Road
Singapore 119077



**Supplementary Figure S1.** Email Chain Between Dr. Crispim and J.Y.J.K. Regarding Correction of Reported Insulin Unit Error.
